# Patella Dislocation with Vertical Axis Rotation: The “Dorsal Fin” Patella

**DOI:** 10.1155/2015/328386

**Published:** 2015-03-25

**Authors:** David Gamble, Quentin Otto, Andrew D. Carrothers, Vikas Khanduja

**Affiliations:** ^1^Trauma and Orthopaedic Department, Addenbrooke's Hospital, Cambridge University Hospitals NHS Foundation Trust, Hill Road, Cambridge CB2 0QQ, UK; ^2^University of Cambridge School of Clinical Medicine, UK

## Abstract

A 44-year-old woman presented following minor trauma to her right knee. While dancing she externally rotated around a planted foot and felt sudden pain in her right knee. She presented with her knee locked in extension with a “dorsal fin” appearance of the soft tissues tented over the patella. This was diagnosed as a rare case of an intraarticular patella dislocation, which was rotated 90 degrees about the vertical axis. Closed reduction in the emergency room was unsuccessful but was achieved in theatre under general anaesthetic with muscle relaxation. Postreduction arthroscopy demonstrated that no osteochondral or soft tissue damage to the knee had been sustained. In patients presenting with a knee locked in extension with tenting of skin over the patella (the “dorsal fin” appearance), intra-articular patella dislocation should be suspected. Attempts to reduce vertical patella dislocations under sedation with excessive force or repeatedly without success should be avoided to prevent unnecessary damage to the patellofemoral joint. In this clinical situation we recommend closed reduction under general anaesthetic followed by immediate knee arthroscopy under the same anaesthetic to ensure that there is no chondral damage to the patella or femoral trochlea and to rule out an osteochondral fracture.

## 1. Introduction

Patella dislocation is an extremely common emergency presentation. Normally the patella is dislocated extra-articularly and almost always laterally, with other types of dislocation being rare. Most spontaneously reduced, but occasionally closed, manipulation is required, which is usually easily achieved in the emergency department. Intra-articular dislocations can be superior or inferior [1] or can be rotated about the horizontal [2] or vertical axis. Dislocation with vertical axis rotation is unusual and 5 times less frequent than the horizontal variant [3]. Relatively few cases have been reported since Cooper first described it in 1844 [4]. We present a rare patient demographic and mechanism of injury for this patient group. We describe a novel treatment technique using immediate arthroscopy to assess the knee joint. This case highlights to clinicians a rare presentation of a dislocated patella with vertical rotation to improve early clinical diagnosis in the early stages of its presentation to ensure correct treatment is provided. It is vital to realise that potential damage can arise either from repeated attempts at reduction or with attempted reduction with excessive force. In addition due to the force from the injury and the rotation of the patella we feel it is imperative to carry out arthroscopy to assess for chondral damage in the acute setting.

## 2. Case Report

**Figure 1:**
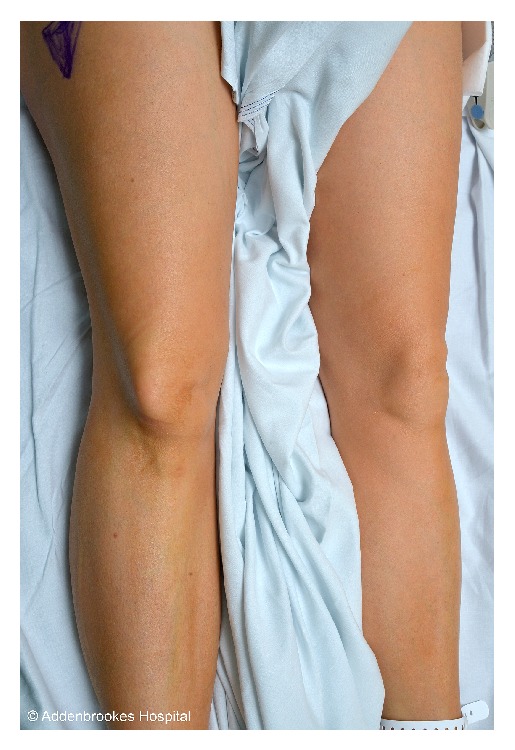
Anterior-posterior view of the patients' knee with the characteristic “dorsal fin” appearance of tenting of the skin over the laterally displaced patella.

**Figure 2:**
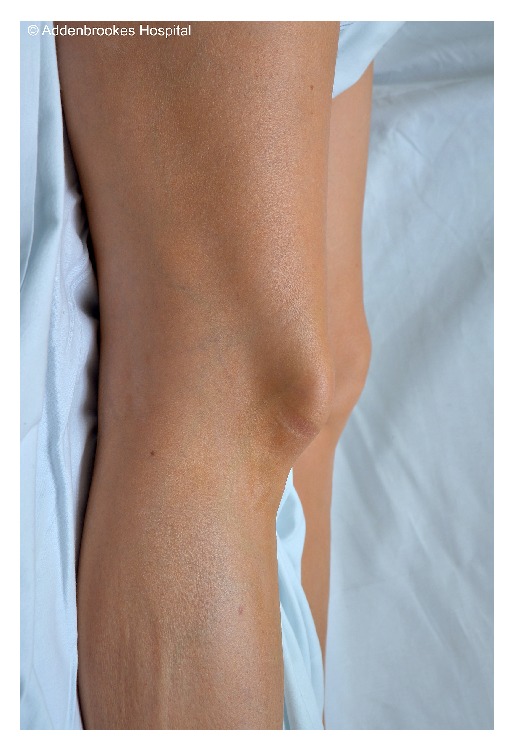
Lateral view of the patients' knee highlighting tenting of the soft tissues over the patella.

A 44-year-old woman presented to the accident and emergency department with acute right knee pain. While dancing the quick-step (an energetic and form-intensive style) she externally rotated about a planted right foot and felt an acute pain in her right knee with her patella shifting laterally. On arrival at hospital her right leg was locked in full extension and she was in severe pain. The patient had no history of previous knee trauma, prior dislocations, or joint hypermobility. She has no other past medical history to suggest any cause for dislocation of the patella. In particular she had no history of paediatric lower limb alignment problems that would predispose her to a dislocated patella. On inspection of the limb there was a deformity of her right patella indicative of a rare patellar dislocation (Figures 1 and 2). The patella appeared rotated on its vertical axis and the skin over the knee joint was tented. It was fixed in position. There was a mild joint effusion and no joint line tenderness. Her range of motion at the right knee was significantly reduced with any flexion from full extension causing the patient significant pain. She was able to straight leg raise on examination indicating that her extensor mechanism was intact. The limb was otherwise neurovascularly intact. The examination of the contralateral limb was entirely normal.

**Figure 3:**
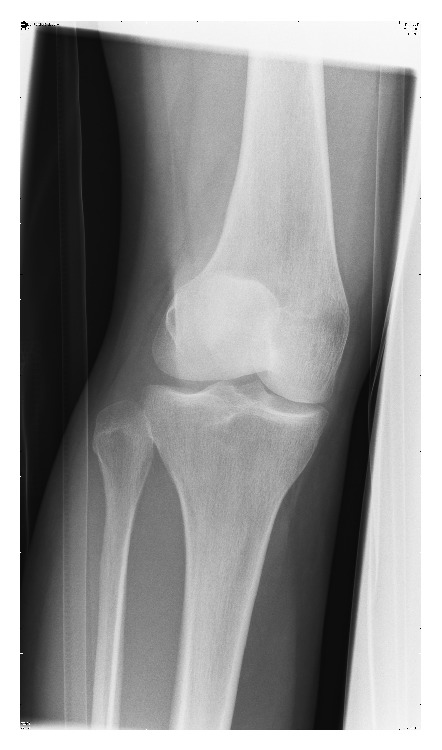
Anterior-posterior radiograph of the knee with a laterally dislocated patella.

**Figure 4:**
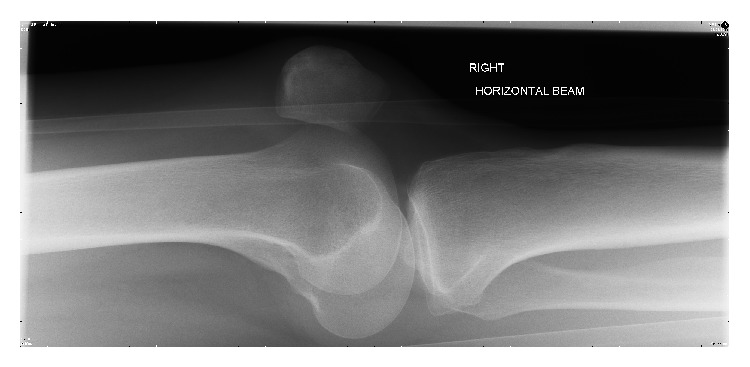
Lateral radiograph of the knee. The knee is held in extension and the patella is rotated 90 degrees.

The AP radiograph revealed a laterally dislocated patella. The lateral radiograph showed an abnormal appearance with the rotated patella impacted in the intercondylar notch of the femur (Figures 3 and 4). In the accident and emergency department one unsuccessful attempt at closed reduction was made under procedural sedation with propofol and fentanyl. The knee joint was fully extended and an attempt was made to manipulate the patella to rotate it back to its normal orientation.

Under a general anaesthetic with muscle relaxant the patella was successfully relocated using a closed technique. The articular surface was facing anterior laterally and it was wedged in the intercondylar notch and impacted into the lateral femoral condyle.

Knowledge of the direction of the articular aspect was vital to the reduction method. With the patient fully muscle relaxed the knee was partially flexed to 30 degrees. Pressure was applied to the medial aspect of the articular surface of the patella that was impacted in the notch. With gentle pressure anteriorly and medially this was pushed up to clear the notch. Once free the natural tension of the extensor mechanism spontaneously reduced the patella. There was no bony injury evident on image intensifier in theatre. The knee was examined under anaesthetic. There was no evidence of any ligamentous injury. There was a normal patellar glide test in assessing for medial and lateral retinaculum damage. The orthopaedic team then conducted an immediate arthroscopy under the same general anaesthetic to check for any osteochondral damage, loose bodies, or soft tissue damage. The arthroscopy findings were of a mild haemarthrosis and mild fibrillation of the lateral trochlear cartilage and lateral patella. There was no gross osteochondral defect and no loose bodies. The medial retinaculum was assessed arthroscopically and was intact. The patient was discharged that day with straight leg immobilisation and orthopaedic followup. Two weeks following her injury she was reviewed in orthopaedic clinic with normal function in her knee. The patient will be followed up at 6 months following the injury. The injury was managed acutely and had normal arthroscopic findings; therefore further investigation with MRI was not felt to be warranted.

## 3. Discussion

This case of patella dislocation with vertical axis rotation is unusual as the mechanism of injury involves minor trauma and involves a 44-year-old female. Cases involving relatively minor trauma have been reported [5–7] but are rare. The literature suggests that cases are predominantly traumatic and commonly occur while playing sport with a direct impact on the knee [5, 6, 8–11]. It can also occur following attempted reduction of a laterally extra-articularly displaced patella [12]. The median age in the literature has been reported as 16 by previous studies [13]. The majority of cases have been reported in younger patients with the hypothesis that ligamentous laxity allows the patella to rotate more freely [14, 15]. Recently reported cases would suggest that the injury is becoming increasingly common in the nonadolescent population [5, 7, 11, 16].

This case showed a successful closed reduction technique and in particular we demonstrate the importance of using muscle relaxant as a vital aid to counteract the quadriceps muscle tension, which often hinders successful closed reduction. The successful management of these dislocations is notoriously difficult to achieve in the accident and emergency department [8, 9, 12, 16–19]. The tension of the quadriceps muscle acts to pull the patella towards the intercondylar notch and makes closed reduction difficult [19]. Although it has been shown to be successfully manipulated in his setting [5–7] the majority of cases required closed reduction under general anaesthetic [11, 16] or open reduction to minimise damage to the knee joint [8, 9, 12, 17–19].

**Figure 5:**
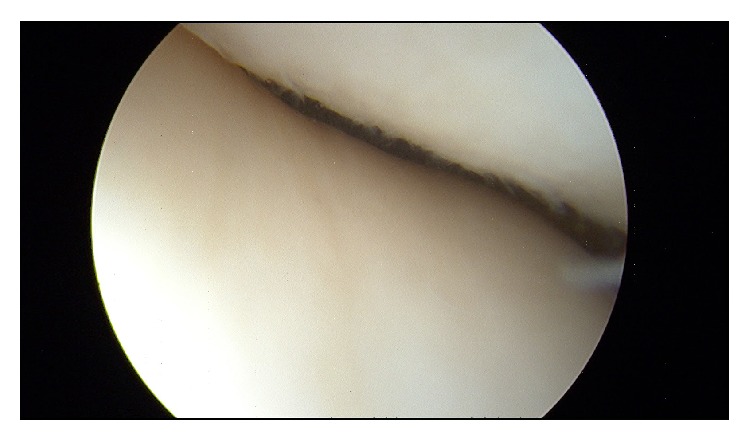
Image from arthroscopy demonstrating no osteochondral damage to the patellofemoral region.

**Figure 6:**
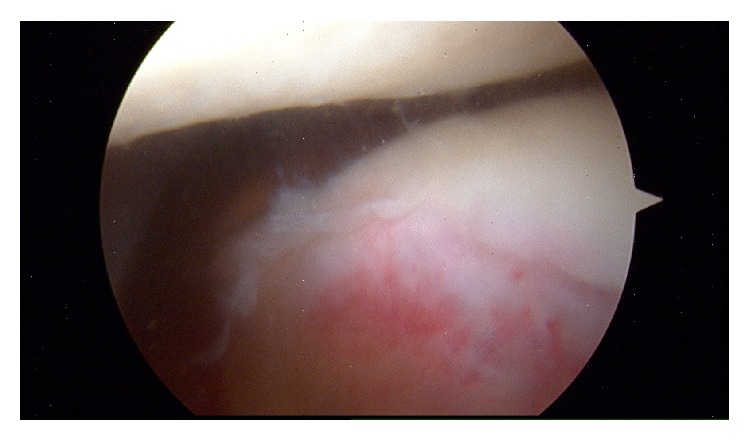
Image from arthroscopy showing fibrillation of the lateral trochlear cartilage.

Immediate postreduction arthroscopy was conducted to assess the degree of underlying damage, guide postoperative recovery, repair any potential damage, and define the long-term prognosis of the knee joint. We demonstrated no significant damage to underlying structures, particularly no osteochondral defects, medial patellofemoral ligament rupture, or damage to the retinaculum (Figures 5 and 6). There are reports of significant soft tissue and osteochondral damage of the knee following this type of injury [12, 17, 18]. This is the first case in the literature to provide postreduction arthroscopic imaging showing that successful reduction can be achieved without damage to the joint. In our case this would be consistent with the relatively trivial mechanism of injury and limited manipulations with inadequate sedation and muscle relaxant. Although of note most cases of reduced patella dislocation are associated with osteochondritis.

The patient in our case presented with the classic sign of a knee locked in extension with characteristic “dorsal fin” appearance of tenting of the anterior soft tissues over the rotated patella. The patella had internally rotated with the articular surface facing laterally. We recommend careful clinical examination and review of the preoperative images to elicit the orientation of the patella. This orientation is the most reported in the literature [7, 9, 12, 16, 17]; however clinical assessment and review of radiographs is essential to prevent injury to the knee while manipulating the patella. An incorrectly manipulated patella could twist the extensor mechanism 180 degrees and risk damage to the soft tissues.

The patient has only been followed up in the acute setting and at the two-week stage and therefore we are unable to comment on the long-term functional outcome from this injury and its management. It would be useful to follow up the long-term results from this patient group to identify if they have any problems with patellar instability or patellofemoral joint degeneration over time.

In a patient presenting with a leg held in extension with tenting of skin over the patella (the “dorsal fin” appearance), intra-articular dislocation with vertical axis rotation should be suspected. Intra-articular dislocations of the patella can be with or without direct trauma and often necessitate manipulation under anaesthetic or even open techniques to achieve good functional reduction and recovery. Attempting to reduce the patella under sedation with excessive force or repeatedly without success could result in unnecessary damage to the patellofemoral joint. We suggest a closed reduction under general anaesthetic with muscle relaxant followed by immediate arthroscopic examination of the knee joint to assess for osteochondral damage.
